# Local radiotherapy for cancer patients is associated with mosaic loss of chromosome Y, a hallmark of male aging

**DOI:** 10.1038/s41514-025-00261-w

**Published:** 2025-07-29

**Authors:** Takuro Kobayashi, Tsuyoshi Hachiya, Yan Lu, Yoshihiro Ikehata, Toshiyuki China, Haruna Kawano, Masayoshi Nagata, Hisamitsu Ide, Shuji Isotani, Shuko Nojiri, Takuro Iwami, Shunsuke Uchiyama, Yasushi Okazaki, Hidewaki Nakagawa, Takayuki Morisaki, Koichi Matsuda, Yoichiro Kamatani, Chikashi Terao, Shigeo Horie

**Affiliations:** 1 Department of Urology, Graduate School of Medicine, Juntendo University, Tokyo, Japan; 2 Data Science and Informatics for Genetic Disorders, Graduate School of Medicine, Juntendo University, Tokyo, Japan; 3 Department of Innovative Longevity, Graduate School of Medicine, Juntendo University, Tokyo, Japan; 4 Department of Medical Technology Innovation Center, Juntendo University, Tokyo, Japan; 5 Laboratory for Statistical and Translational Genetics, RIKEN Center for Integrative Medical Sciences, Yokohama, Kanagawa Japan; 6 Diagnostics and Therapeutics of Intractable Diseases, Intractable Disease Research Center, Graduate School of Medicine, Juntendo University, Tokyo, Japan; 7 Laboratory for Cancer Genomics, RIKEN Center for Integrative Medical Sciences, Kanagawa, Japan; 8 BioBank Japan, Institute of Medical Science, University of Tokyo, Tokyo, Japan; 9 Department of Computational Biology and Medical Sciences, Laboratory of Clinical Genome Sequencing, Graduate School of Frontier Sciences, The University of Tokyo, Tokyo, Japan; 10 Laboratory of Genome Technology, Human Genome Center, Institute of Medical Science, The University of Tokyo, Tokyo, Japan; 11 Department of Computational Biology and Medical Sciences, Laboratory of Complex Trait Genomics, Graduate School of Frontier Sciences, The University of Tokyo, Tokyo, Japan; 12 Clinical Research Center, Shizuoka General Hospital, Shizuoka, Japan; 13 The Department of Applied Genetics, The School of Pharmaceutical Sciences, University of Shizuoka, Shizuoka, Japan; 14 Present Address: Hematovascular Biology Center, Robert M. Berne Cardiovascular Research Center, University of Virginia School of Medicine, Charlottesville, VA USA

**Keywords:** Cancer, Genetics, Diseases, Medical research, Risk factors

## Abstract

Mosaic loss of chromosome Y (mLOY) is the most common somatic mutation in hematopoietic cells of aging men and is linked to cancer risk and mortality. However, its relationship with treatment modalities remains unclear. In 348 prostate cancer patients at Juntendo University Hospital, local radiotherapy was associated with a higher prevalence of mLOY (OR = 2.55, 95% CI: 1.08–6.50, *P* = 0.04), whereas surgery and endocrine therapy were not. We then examined BioBank Japan data from over 30,000 patients with prostate, lung, colorectal, and gastric cancers. Fixed-effect meta-analysis across these sites showed a significant association between radiotherapy and mLOY (OR = 1.48, 95% CI: 1.11–1.98, *P* = 0.01). No significant effect heterogeneity was observed across cancer types (Q = 0.36, *I*² = 0%, *P* = 0.95). Our findings suggest that radiotherapy may exacerbate genomic instability, indicating a potential vulnerability in certain cancer patients to DNA damage induced by radiation therapy.

## Introduction

Somatic mutations are observed in non-cancerous human cells, and range from single-nucleotide substitutions to an increase, or loss of entire chromosomes^1^. Mosaic loss of chromosome Y (mLOY) is the most frequently observed chromosome-level somatic mutation in hematopoietic cells of aging men^2–4^. Smoking is an accelerated aging factor associated with mLOY^5^, and recent studies have also shown that mLOY is associated with an increased risk of various age-related diseases such as cardiovascular disease^6,7^, Alzheimer’s disease^8^, chronic kidney disease^9^, and cancer^6,10–15^; so, mLOY could be considered as a possible biomarkers of aging^16,17^.

Emerging evidence from multiple studies have shown robust associations between mLOY with cancer incidence and prognosis^6,10,11,13,18^. A prospective study in which data from the UK biobank were analyzed (>200,000 men free from cancer at baseline, and >13,000 incident solid tumor cases during follow-up), showed that mLOY was moderately associated with cancer incidence^13^. In analyses from the cancer genome-wide association studies (GWAS), a significant association was found between mLOY and non-hematological cancer in three prospective cohorts (>8500 cancer cases, and >5000 cancer-free controls)^10^. In the Uppsala Longitudinal Study of Adult Men (ULSAM), a cohort of approximately 1000 elderly men, free from a cancer diagnosis at baseline, mLOY was significantly associated with more than a 3-fold higher cancer-specific mortality^11^. A survival analysis using data from the Biobank Japan Project (>95,000 male participants at baseline) revealed an association between higher mLOY signal levels and increased hazard ratios for all-cause mortality^18^.

Interestingly, data from three prospective cohort studies have suggested that the association between detectable mLOY and cancer was more evident at, and after cancer diagnosis (odds ratio [OR], 1.52; 95% confidence interval [CI], 1.23–1.87) compared to one year before cancer diagnosis (OR, 1.19; 95% CI, 1.00–1.42)^10^. These findings are corroborated with data from a longitudinal study that indicated the proportion of blood cells lacking the Y chromosome was markedly increased after cancer diagnosis compared to before a cancer diagnosis^11^. Taking this into account, we hypothesized that cancer treatment may enhance the progression of mLOY. However, the relationship between cancer treatment and mLOY has been poorly investigated.

In this study, we investigated the relationship between treatment modalities and mLOY among 348 prostate cancer patients, followed by the replication analysis of the finding among 10,672 prostate, 4437 lung, 8213 colorectal, and 7524 gastric cancer patients.

## Results

### Prevalence of mLOY and clinical characteristics in the Juntendo prostate cancer patients

**Fig. 1:**
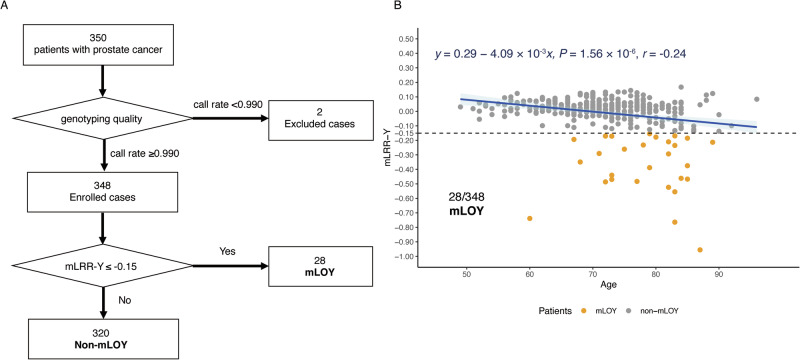
Data analysis flow of the Juntendo dataset. **A** Data analysis flow of the Juntendo cohort, illustrating the selection of 348 patients included in the analysis, of whom 28 (8.0%) were classified as having mLOY. **B** Scatter plot showing the relationship between age and mLRR-Y values. The dashed line represents the mLOY threshold (mLRR-Y ≤ −0.15), while orange dots indicate patients classified as having mLOY. Abbreviations: mLRR-Y median of the log R ratio of probes in the male-specific region of chromosome Y, mLOY mosaic loss of chromosome Y.

A total of 350 patients with prostate cancer were initially enrolled in the study (Fig. 1A). Two patients were excluded due to low-quality fluorescence intensity data, leaving 348 patients for the final analysis. Among these, 28 patients (8.0%) had mLOY defined as mLRR-Y ≤ −0.15. A significant inverse correlation between age and mLRR-Y was observed (Fig. 1B; *y* = 0.29 − 4.09 × 10⁻³*x*, *P* = 1.56 × 10⁻⁶), as consistent with previous studies^17^. The clinical characteristics, including iPSA, Gleason score, and T stage, were not significantly different between mLOY and non-mLOY groups according to the Fisher’s exact test (Table 1).

**Table 1:** Baseline characteristics by mLOY status in the Juntendo prostate cancer patients

Characteristic	mLOY, N(%)	non-mLOY, N(%)	*P* value
Subjects	28 (100.0)	320 (100.0)	–
iPSA			0.82
<10 ng/mL	16 (57.1)	191 (59.7)	–
10–19.9 ng/mL	7 (25.0)	64 (20.0)	–
≥20.0 ng/mL	5 (17.9)	57 (17.8)	–
Gleason score			0.14
≤6	6 (21.4)	81 (25.3)	–
7	8 (28.6)	138 (43.1)	–
≥8	13 (46.4)	89 (27.8)	–
T stage			0.92
T1	4 (14.3)	41 (12.8)	–
T2	18 (64.3)	202 (63.1)	–
≥T3	6 (21.4)	76 (23.8)	–

*iPSA* initial prostate-specific antigen, *mLOY* mosaic loss of chromosome Y.

### Treatment modalities and mLOY in the Juntendo prostate cancer patients

**Fig. 2:**
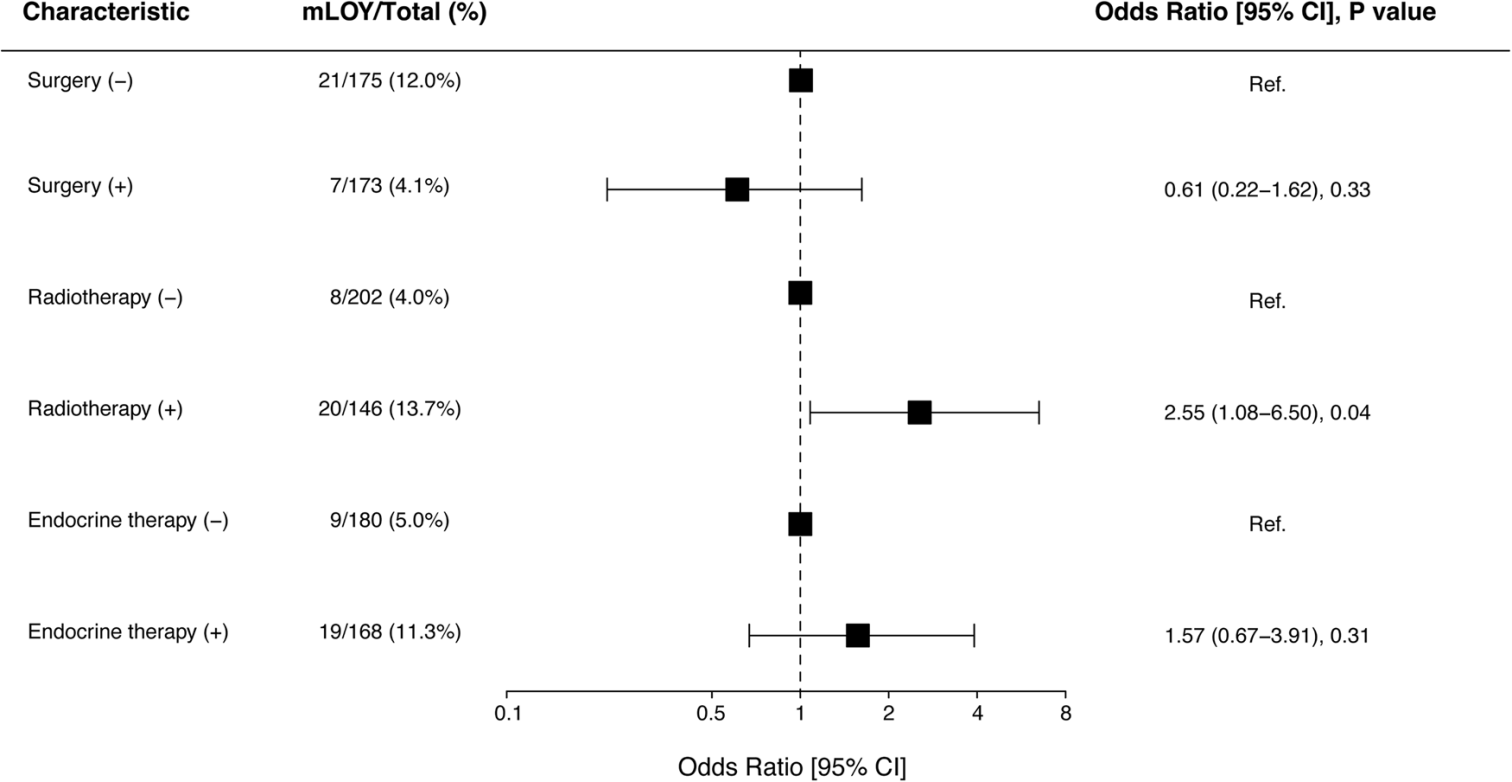
Associations between clinical characteristics and mLOY in the Juntendo prostate cancer patients. Abbreviations: CI confidence interval, mLOY mosaic loss of chromosome Y, OR odds ratio.

We investigated the potential association between treatment modalities, including surgery, local radiotherapy, and endocrine therapy, and the prevalence of mLOY. A significant association was observed between local radiotherapy and mLOY, with an OR of 2.55 (95% CI: 1.08–6.50; *P* = 0.04) (Fig. 2). Patients who experienced local radiotherapy had a higher prevalence of mLOY (13.7%) compared to those who did not (4.0%). Endocrine therapy (11.3% vs. 5.0%; OR = 1.57; 95% CI: 0.67–3.91; *P* = 0.31) and surgical treatment (4.1% vs. 12.0%; OR = 0.61; 95% CI: 0.22–1.62; *P* = 0.33) were not significantly associated with mLOY.

### Replication analysis in the Biobank Japan cancer patients

**Fig. 3:**
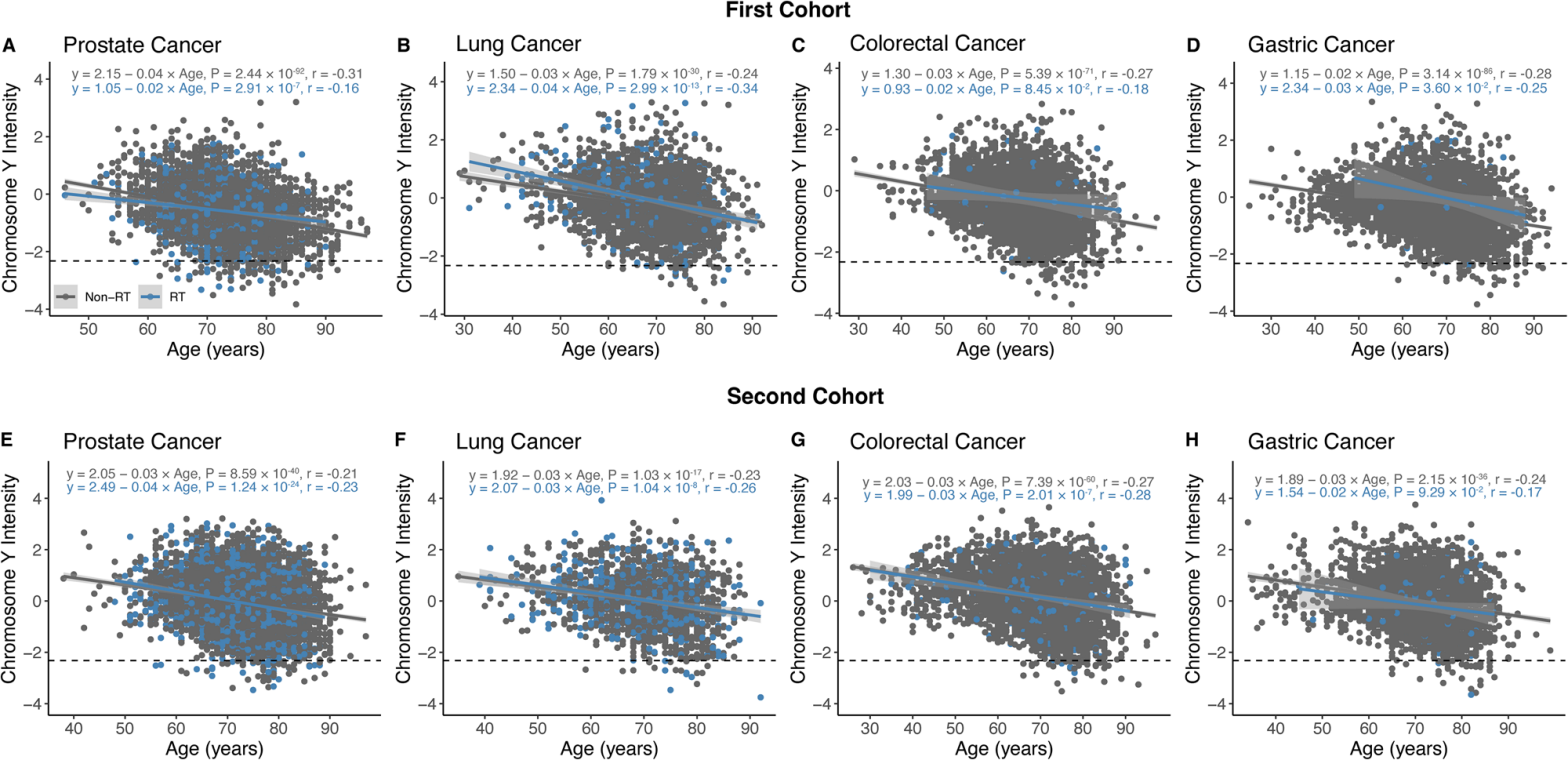
Relationship between age and intensity signal of chromosome Y in the Biobank Japan dataset. Scatter plots for the first cohort, illustrating the relationship between age and chr Y intensity in prostate cancer (**A**), lung cancer (**B**), colorectal cancer (**C**), and gastric cancer (**D**). Scatter plots for the second cohort, depicting the same relationships in prostate cancer (**E**), lung cancer (**F**), colorectal cancer (**G**), and gastric cancer (**H**). The dashed line represents the threshold for the mLOY signals, defined as the top 1% of individuals with the lowest mLRR-Y. Gray and blue dots indicate patients who did not receive radiotherapy (non-RT) and those who did (RT), respectively. Regression lines for non-RT (gray) and RT (blue) groups are shown, with corresponding equations and P-values.

In the Biobank Japan (BBJ) first and second cohorts, a consistent significant negative correlation between age and mLOY signal was observed across four cancer sites (prostate, lung, colorectal, and gastric) in both the first and second cohorts (Fig. 3). The prevalence of mLOY in the first cohort was highest in prostate cancer (2.14%), followed by colorectal cancer (1.28%), lung cancer (1.21%), and gastric cancer (1.08%) (Table 2). Among patients who received local radiotherapy, mLOY prevalence was markedly higher in prostate cancer (21.1%) and lung cancer (21.9%) compared to colorectal cancer (1.82%) and gastric cancer (1.92%). In the second cohort, the overall prevalence of mLOY was highest in gastric cancer (1.28%), followed by prostate cancer (1.24%), lung cancer (1.11%), and colorectal cancer (1.05%). Among patients receiving local radiotherapy, the prevalence of mLOY was markedly higher in prostate cancer (34.8%) and lung cancer (35.0%), compared to colorectal cancer (9.76%) and gastric cancer (5.71%).

**Table 2:** Characteristics of prostate, lung, colorectal, and gastric cancer patients in Biobank Japan

Characteristic	Prostate	Lung	Colorectal	Gastric
First cohort				
Subjects, N	5090	2637	4303	4793
Age (mean ± SD)	72.8 ± 6.9	67.8 ± 9.3	67.7 ± 9.0	67.2 ± 9.7
Local radiotherapy, N (%)	925 (18.2)	426 (16.2)	98 (2.3)	69 (1.4)
mLOY, N (%)	109 (2.14)	32 (1.21)	55 (1.28)	52 (1.08)
mLOY in RT, N (%)	23 (21.1)	7 (21.9)	1 (1.82)	1 (1.92)
Second cohort				
Subjects, N	5582	1800	3910	2731
Age (mean ± SD)	72.4 ± 7.5	69.7 ± 9.8	68.2 ± 10.4	70.5 ± 9.1
Local radiotherapy, N (%)	1848 (33.1)	451 (25.1)	325 (8.3)	99 (3.6)
mLOY, N (%)	69 (1.24)	20 (1.11)	41 (1.05)	35 (1.28)
mLOY in RT, N (%)	24 (34.8)	7 (35.0)	4 (9.76)	2 (5.71)

*mLOY* mosaic loss of chromosome Y, *RT* radiotherapy, *SD* standard deviation.

**Fig. 4:**
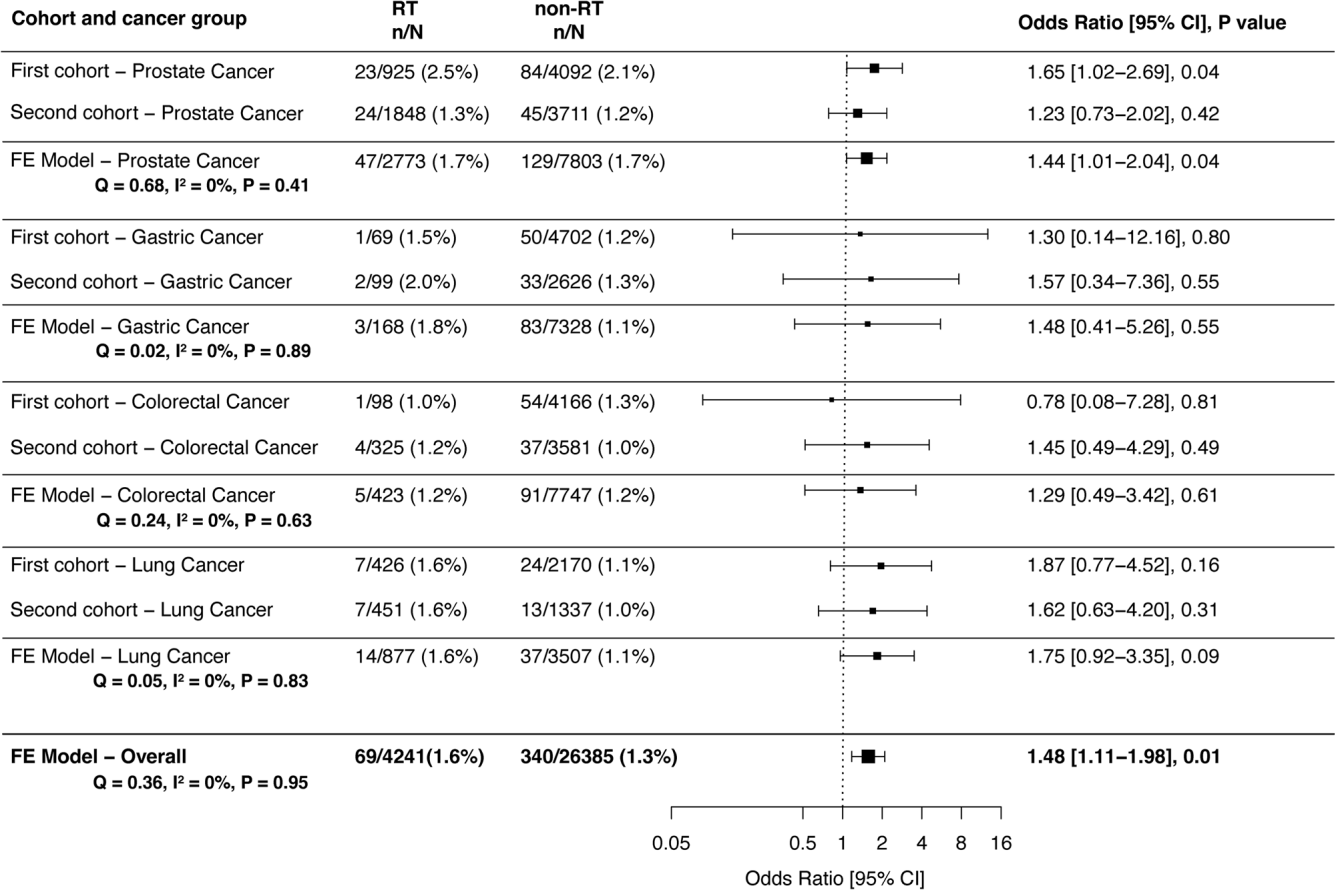
Meta-analysis of associations between local radiotherapy and mLOY in the Biobank Japan dataset. Abbreviations: CI confidence interval, FE fixed-effect model, mLOY mosaic loss of chromosome Y, RT radiotherapy.

We investigated the association between local radiotherapy and mLOY in the BBJ dataset. Meta-analysis across BBJ first and second cohorts were performed for each cancer site (prostate, lung, colorectal, and gastric cancers) (Fig. 4). Among prostate cancer patients, the meta-analysis showed a significant association between local radiotherapy and mLOY, with an OR of 1.44 (95% CI: 1.01–2.04, *P* = 0.04). The heterogeneity was low (Q = 0.68, *I*² = 0%, *P* = 0.41), indicating consistent OR estimates across the two cohorts.

Among lung, colorectal, and gastric cancer patients, the meta-analysis suggested a positive but non-significant association and no effect heterogeneity was observed (Fig. 4).

The overall meta-analysis across the four cancer sites showed a significant association between local radiotherapy and mLOY, with an OR of 1.48 (95% CI: 1.11–1.98, *P* = 0.01). No significant effect heterogeneity was observed across cancer types (Q = 0.36, *I*² = 0%, *P* = 0.95).

### Stratified analysis by time since radiotherapy

To evaluate the potential time-dependent effects of radiotherapy on mLOY, we stratified patients by the interval between radiotherapy completion and DNA sample collection.

In the Juntendo cohort, patients were divided into three groups: those who received radiotherapy <1 year prior to sampling, those who received radiotherapy ≥1 year prior, and those who did not receive radiotherapy (reference group). Patients who received radiotherapy ≥1 year before sampling had an OR of 2.45 (95% CI: 1.00–6.41, *P* = 0.06), while those who received radiotherapy within <1 year had an OR of 3.11 (95% CI: 0.63–12.15, *P* = 0.12). There was no statistically significant difference between these two estimates (p for heterogeneity = 0.79).

In the BBJ cohort, fixed-effect meta-analysis across four cancer types (prostate, lung, colorectal, and gastric cancers) revealed that radiotherapy <1 year prior to sample collection was significantly associated with increased mLOY (OR = 1.66, 95% CI: 1.18–2.32, *P* = 0.0034). In contrast, radiotherapy ≥1 year prior showed no significant association (OR = 1.38, 95% CI: 0.90–2.13, *P* = 0.14). However, the difference in effect sizes between these two subgroups did not reach statistical significance (p for heterogeneity = 0.51).

### Sensitivity analysis using alternative mLOY thresholds

To address concerns regarding differing mLOY definitions across cohorts, we performed a sensitivity analysis using a range of mLOY thresholds, defined by the top 1% to 50% of individuals with the most extreme Y chromosome loss (i.e., lowest mLRR-Y values).

In the Juntendo cohort, we standardized the mLRR-Y values following the same approach as used in the UK Biobank, and calculated odds ratios for each percentile cutoff. To ensure statistical reliability, thresholds with fewer than 10 mLOY events in the radiotherapy group were excluded. The strongest associations were observed in the top 5–10%, consistent with our original threshold (approximately top 8%, mLRR-Y ≤ –0.15, Supplementary Fig. 1). It should be noted that, prior to examining the results with varying mLRR-Y, we followed previous studies and conducted the analysis of the Juntendo dataset using a threshold of mLRR-Y ≤ −0.15^6,19–21^.

In the BBJ cohort, we observed a similar pattern. At more extreme thresholds (top 1–5%), radiotherapy was significantly associated with mLOY. At broader thresholds (top 20% and beyond), the association weakened or reversed (Supplementary Fig. 2).

### Modality-specific analysis of radiotherapy type

Given the differing patterns of bone marrow exposure across radiotherapy modalities, we conducted an additional analysis in the Juntendo cohort by separating patients based on treatment type: intensity-modulated radiotherapy (IMRT), brachytherapy (Seed Implantation), and proton therapy. Due to the limited number of proton therapy cases (n = 4), they were excluded from the regression analysis. Compared to patients without radiotherapy, those treated with IMRT (n = 110) had a significantly higher odds of mLOY (OR = 3.05, 95% CI: 1.26–7.95, *P* = 0.016). In contrast, brachytherapy (n = 32) was not significantly associated with mLOY (OR = 1.46, 95% CI: 0.30–5.61, *P* = 0.60).

While we were unable to obtain full dose-volume histograms, we collected available total dose data in the Juntendo cohort. IMRT patients typically received 72 Gy (mean: 72.1 Gy), whereas brachytherapy patients received higher nominal doses (mean: 157.7 Gy). To further explore the dose–response relationship, we performed a logistic regression analysis restricted to IMRT patients, in whom radiation was uniformly delivered to the pelvic region. This analysis revealed a statistically significant association between total radiation dose and the presence of mLOY. Specifically, each 1 Gy increase in radiation dose was associated with a 1.4% increase in the odds of mLOY (OR = 1.014, 95% CI: 1.002–1.027, P = 0.024). Among brachytherapy patients, no statistically significant association was observed (OR = 1.003, 95% CI: 0.993–1.011, P = 0.58). The difference in effect sizes between the two modalities was not statistically significant (p for heterogeneity = 0.16).

## Discussion

This study, to the best of our knowledge, is the first to report a significant association between local radiotherapy and the prevalence of mLOY among cancer patients. Among the Juntendo prostate cancer patients, local radiotherapy was significantly associated with an increased prevalence of mLOY, whereas surgery and endocrine therapy was not. The significant association between local radiotherapy and mLOY was confirmed in the BBJ follow-up analysis incorporating 10,672 prostate, 4437 lung, 8213 colorectal, and 7524 gastric cancer patients, with no significant effect heterogeneity across cancer sites.

Recent studies suggest that mLOY may not be merely a biomarker of genomic instability but could also contribute directly to the development of pathological conditions. Sano et al. showed using a murine model that mLOY in hematopoietic cells causally contributes to age-related diseases, including fibrosis and reduced cardiac function, by driving macrophage polarization toward a profibrotic phenotype. Notably, treatment with a transforming growth factor β1 (TGF-β1)–neutralizing antibody ameliorated cardiac dysfunction in these mLOY models, highlighting a potential therapeutic target^7^. Their study also showed that human patients with mLOY in blood are at an increased risk of cardiovascular disease and heart failure–associated mortality. These findings highlight the detrimental impact of mLOY on systemic health.

Although molecular mechanisms underlying the etiology of mLOY have yet to be fully elucidated, recent genetic studies suggest that mLOY may arise in hematopoietic stem and progenitor cells (HSPCs)^4,18^. Genome-wide association studies have revealed that most mLOY-associated genetic variants were located within, or near cell cycle genes that are involved in DNA synthesis, damage response, mitosis, and apoptosis^4,10,18,22^. Studies have also shown that fine-mapped mLOY-associated variants were enriched in genomic regions, accessible in HSPCs, but not in other cell types^4,18^. These data suggest that mLOY-associated variants exert their effects in HSPCs, rather than white blood cells, which are more differentiated.

The mechanisms by which local radiotherapy, primarily targeting solid tumors, leads to the detection of mLOY in peripheral blood cells are not fully understood. One possible explanation involves the direct irradiation of hematopoietically active bone marrow during treatment. In cancers such as prostate and lung cancer, radiotherapy often includes anatomical regions rich in active bone marrow—such as the pelvis, ribs, sternum, and thoracic and lumbar spine—which together account for the majority of adult hematopoiesis^23–26^. Clinical studies have shown that radiation exposure to these regions is associated with measurable declines in neutrophils, lymphocytes, and platelets. In prostate and gynecologic cancers, pelvic radiotherapy correlates with increased risk of acute hematologic toxicity in a dose-dependent manner^23,24^. Similarly, in lung and esophageal cancer patients, increased radiation dose to the thoracic vertebral bodies, sternum, and ribs has been associated with grade ≥2 hematologic toxicity, including leukopenia and neutropenia^25,26^.

Ionizing radiation activates key kinases such as ataxia telangiectasia mutated (ATM) and ataxia telangiectasia and Rad3 related (ATR), triggering a DNA damage response that promotes cell cycle arrest and senescence^27–29^. These effects on HSPCs may promote genomic instability, including the loss of the Y chromosome.

Stratified analysis in both the Juntendo and BBJ cohorts showed that patients who had undergone radiotherapy within the past year exhibited slightly higher odds ratios for the association with mLOY compared to those treated earlier, although the heterogeneity of the effect was not statistically significant. Further studies are needed to determine whether the impact of radiotherapy on mLOY attenuates over time following treatment.

Sensitivity analyses with varying mLOY thresholds in both cohorts consistently showed stronger associations between radiotherapy and mLOY. In the Juntendo cohort, odds ratios increased progressively as the definition was restricted to individuals with the highest degree of Y chromosome loss. The association peaked within the top 5–10%, which was consistent with the original cutoff used in this study. In the BBJ cohort, a similar trend was observed, with higher odds ratios at the most extreme thresholds (top 1–5%), while broader definitions resulted in gradually decreasing associations.

In addition, a modality-specific analysis within the Juntendo cohort explored the dose–response relationship between radiation exposure and mLOY. Among IMRT-treated patients, a statistically significant association between total radiation dose and the presence of mLOY was observed, even within a relatively narrow dose range. In this study, no significant increase in mLOY was observed in the group treated with brachytherapy. In contrast to external beam radiation therapy, brachytherapy delivers radiation internally to the target without traversing overlying tissues, and the irradiated volume is substantially smaller^30^. Clinically, hematologic toxicity has been reported to be minimal^31^, suggesting that radiation-induced bone marrow damage is unlikely to contribute substantially to the risk of mLOY in this setting.

This study has several limitations that should be acknowledged. First, since this is an observational study, causal relationships between local radiotherapy and mLOY cannot be established. Second, this study used different thresholds for defining mLOY and different sample sources across datasets. mLOY was defined as mLRR-Y ≤ −0.15 for the Juntendo dataset, whereas mLOY was defined as the top 1% of standardized mLRR-Y for the BBJ dataset. Saliva samples were used to assess mLOY in the Juntendo dataset, whereas blood samples were used in the BBJ dataset. Saliva contains both buccal epithelial cells and leukocytes^32^, and therefore, clonal expansion of HSPCs lacking the Y chromosome could be detected using saliva samples^2,33^.

In conclusion, this study found a significant association between local radiotherapy and mLOY among cancer patients. Our findings suggest that radiotherapy may exacerbate genomic instability by disrupting the balance between DNA damage and the DNA repair response, indicating a potential vulnerability in certain cancer patients to DNA damage induced by radiation therapy. Identifying such vulnerabilities could inform personalized treatment strategies, potentially mitigating the adverse effects of radiotherapy. Future research is required to assess the long-term clinical consequences of increased mLOY following radiotherapy.

## Methods

### Study design and population

This study utilized data from two independent datasets. The first dataset comprised 350 patients with prostate cancer who had not progressed to castration-resistant prostate cancer, enrolled at Juntendo University Hospital, Tokyo, between March 2018 and July 2020. Clinical variables included initial prostate-specific antigen (iPSA) levels, Gleason scores (GS), tumor T stage, and treatment modalities. Prostate cancer was diagnosed by biopsy, with histopathological evaluations performed by skilled pathologists. Cases referred from external institutions were re-evaluated when possible. iPSA levels were determined from blood samples collected before diagnosis, and GS was graded from 2 to 10 using the Gleason grading system^34^. Tumor staging was classified according to the TNM 1992 classification^35,36^. Endocrine therapy consisted of luteinizing hormone-releasing hormone (LH-RH) agonists, LH-RH antagonists or anti-androgens, either as single agents or in combination. Surgical treatment consisted of radical retropubic prostatectomy or robotic-assisted radical prostatectomy, including prostatectomy in conjunction with total cystectomy. Local radiation therapy included IMRT, brachytherapy, proton therapy, and salvage radiation after local recurrence. Palliative irradiation for patients with bone metastases was not included because it was noncurative irradiation. Chemotherapy was excluded from the analysis due to the limited number of patients, including docetaxel and cabazitaxel (*n* = 28). Therefore, the relationship between chemotherapy and mLOY could not be assessed. Data for the treatment method were defined at the time of sample collection for mLOY analysis. All patients were categorized according to treatment modalities (including radiotherapy, endocrine therapy, and surgery) that had already been initiated or administered prior to the collection of DNA samples.

The second dataset was derived from the BBJ project, a multicenter hospital-based registry designed to collect clinical and genomic data on various diseases^37,38^. This dataset consisted of two independent cohorts: first cohort and second cohort, with distinct enrollment periods and disease coverage. The first cohort included approximately 200,000 patients enrolled between June 2003 and March 2008, with baseline data comprising DNA, serum, and clinical information for 47 diseases. For patients who continued hospital visits after April 2008, additional serum and clinical data were collected. The second cohort included approximately 70,000 patients enrolled between December 2012 and December 2017, with baseline data comprising DNA and clinical information for 38 diseases, 34 of which overlapped with the first cohort, along with four additional diseases. This study targeted male cancer patients with available mLOY data. The first cohort included prostate cancer (*n* = 5090), gastric cancer (*n* = 4793), colorectal cancer (*n* = 4303), lung cancer (*n* = 2637), esophageal cancer (*n* = 1094), liver cancer (*n* = 245), blood cancer (*n* = 90), pancreatic cancer (*n* = 46), and breast cancer (*n* = 8). The second cohort comprised prostate cancer (*n* = 5582), colorectal cancer (*n* = 3910), gastric cancer (*n* = 2731), lung cancer (*n* = 1800), esophageal cancer (*n* = 717), liver cancer (*n* = 120), blood cancer (*n* = 59), pancreatic cancer (*n* = 35), and breast cancer (*n* = 6). Clinical information for both cohorts was collected through standardized questionnaires or medical record surveys at the time of enrollment. Patients were also categorized according to treatment modalities that had already been initiated or administered prior to the collection of DNA samples.

This study was conducted with the approval by the Institutional Review Board (IRB) of Juntendo University Hospital (IRB #M18-0234), and written informed consent was obtained from all participants. This study was performed in accordance with the principles expressed in the Declaration of Helsinki 2013.

### Definition of mLOY

Genotyping microarray fluorescence intensity data were processed separately for each dataset. In the Juntendo dataset, DNA was extracted from saliva samples and analyzed using the Infinium CoreExome-24+ kit. Samples with missing intensity data in more than 1% of probes specific to the male-specific region of the Y chromosome were excluded from the analysis. For the BBJ dataset, DNA was extracted from peripheral blood samples and analyzed using multiple genotyping platforms, including Human OmniExpressExome and Human Exome arrays. Samples with a missing call rate exceeding 5% were excluded.

In the Juntendo dataset, normalized and log-transformed fluorescence intensity data were represented as the “log R ratio”^11^. Based on thresholds in previous studies, mLOY was defined as median of the log R ratio of probes in the male-specific region of chromosome Y (mLRR-Y) ≤ −0.15^6,19–21^.

In the BBJ dataset, the microarray platforms used for data collection differed between the first and second cohorts, making direct comparison of absolute mLRR-Y values across cohorts biased. To address this issue, and in accordance with previous studies, mLOY was defined using a relative threshold based on percentiles within each cohort^18^. Specifically, individuals in the top 1% of mLOY signal distribution (i.e., those with the lowest mLRR-Y) were classified as having mLOY. This approach ensured consistency across the two BBJ cohorts, accounting for platform-specific differences in microarray fluorescence intensity.

### Statistical analysis

Differences in the distribution of clinical background factors (iPSA levels, Gleason score, and T stage) between mLOY and non-mLOY groups were assessed using Fisher’s exact test in the Juntendo dataset. Fisher’s exact test was chosen due to its suitability for small sample sizes and categorical data. These analyses aimed to evaluate whether the prevalence of mLOY varied significantly across baseline characteristics and to identify potential confounders for subsequent analyses.

Logistic regression models were used to evaluate associations between treatment modalities and mLOY. For the Juntendo dataset, models were adjusted for age at sample collection and smoking status. For the BBJ dataset, the replication analysis was conducted using logistic regression models adjusted for age and smoking status separately for cancer sites as well as for the first cohort and second cohort. We restricted cancer sites with sufficient number of patients (*n* > 1000). As the result, four cancer sites (prostate, lung, colorectal, and gastric) were evaluated. After separately analyzing the two cohorts for each cancer type, a fixed-effect meta-analysis across the first and second cohorts was performed, followed by a fixed-effect meta-analysis among cancer sites. Heterogeneity among studies was assessed using Cochran’s Q test, I² statistic, and the p-value for heterogeneity. Cochran’s Q test evaluates whether observed differences in effect sizes across studies are greater than expected by chance, with the p-value indicating the statistical significance of heterogeneity^39^. The I² statistic quantifies the proportion of total variation attributable to heterogeneity, with thresholds of <25%, 25%–75%, and >75% indicating low, moderate, and high heterogeneity, respectively^40^.

All statistical analyses were conducted using R software (version 4.4.0, R Foundation for Statistical Computing, Vienna, Austria). Statistical significance was defined as two-tailed *P* < 0.05.

## Supplementary information

Supplementary Information

## Data Availability

Data supporting the findings of the Juntendo dataset are available from the corresponding author upon reasonable request. Genotyping data for the Biobank Japan datasets are available from the Japanese Genotype-phenotype Archive (JGA; http://trace.ddbj.nig.ac.jp/jga/index_e.html) by application with accession code JGAD00000000123. The custom R scripts and analysis pipelines used in this study are available from the corresponding author upon reasonable request.
