# Supplementary material for: Local radiotherapy for cancer patients is associated with mosaic loss of chromosome Y, a hallmark of male aging
Source: NPJ Aging. 2025 Jul 29;11(1):69. doi: 10.1038/s41514-025-00261-w (PMC12307641; doi:10.1038/s41514-025-00261-w)

Local radiotherapy for cancer patients is associated with mosaic loss of chromosome Y, a hallmark of male aging

Takuro Kobayashi[1,2,3], Tsuyoshi Hachiya[2,4,*], Yan Lu[1], Yoshihiro Ikehata[1,2], Toshiyuki China[1], Haruna Kawano[1,2], Masayoshi Nagata[1], Hisamitsu Ide[1,4], Shuji Isotani[1], Shuko Nojiri[5], Takuro Iwami[6], Shunsuke Uchiyama[6], Yasushi Okazaki[7], Hidewaki Nakagawa[8], Takayuki Morisaki[9,10], Koichi Matsuda[9,10,11], Yoichiro Kamatani[6,12], Chikashi Terao[6,13,14], Shigeo Horie[1,2,4†,*]

Supplementary Figure 1. Association between radiation therapy and top N% mLOY in Juntendo cohort.

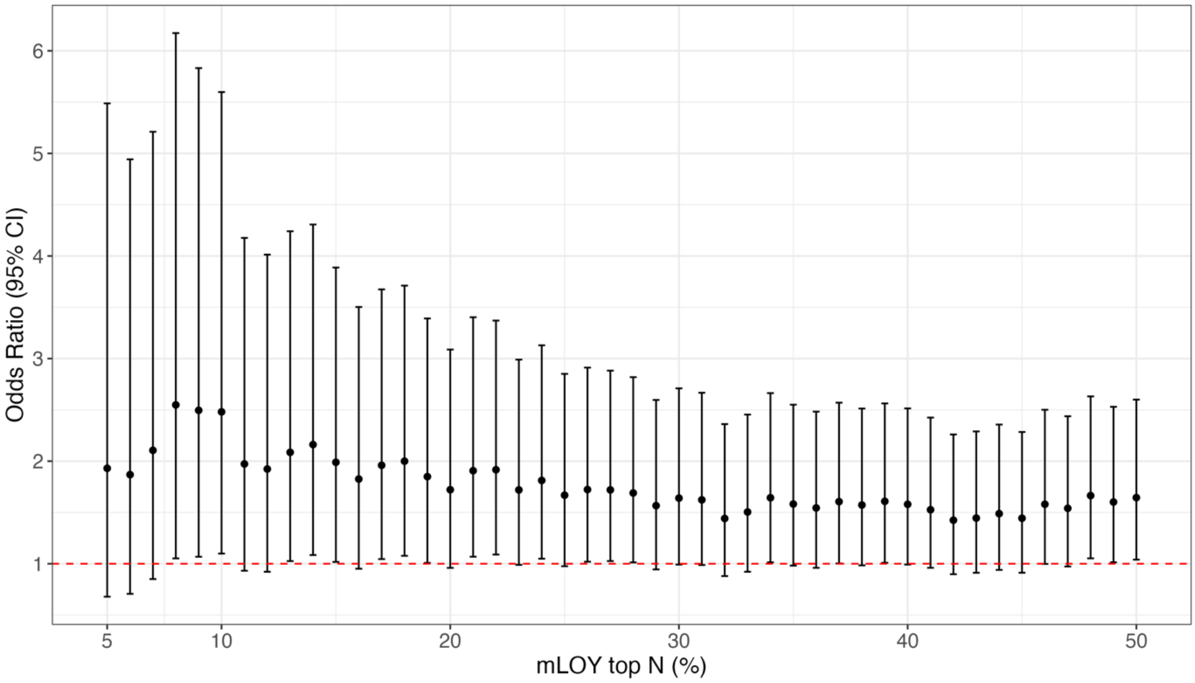

Supplementary Figure 2. Overall association between radiation therapy and top N% mLOY in Biobank Japan cohort.

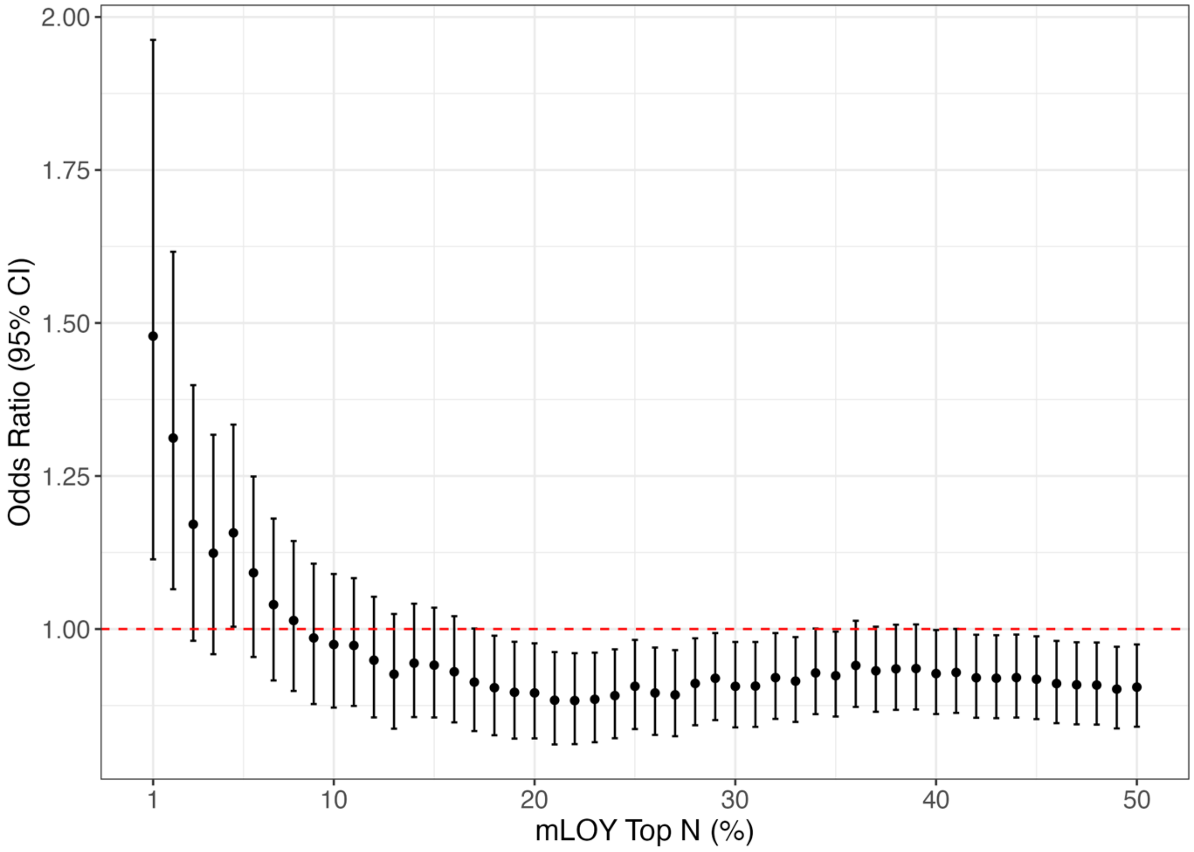

Supplement: Supplementary file 1 — Supplementary Information [file 41514_2025_261_MOESM1_ESM.pdf]
